# Clinician treatment choices for post-traumatic stress disorder: ambassadors survey of psychiatrists in 39 European countries

**DOI:** 10.1192/j.eurpsy.2024.19

**Published:** 2024-03-07

**Authors:** Martina Rojnic Kuzman, Frank Padberg, Benedikt L. Amann, Meryam Schouler-Ocak, Zarko Bajic, Tarja Melartin, Adrian James, Julian Beezhold, Jordi Artigue Gómez, Celso Arango, Tihana Jendricko, Jamila Ismayilov, William Flannery, Egor Chumakov, Koray Başar, Simavi Vahip, Dominika Dudek, Jerzy Samochowiec, Goran Mihajlovic, Fulvia Rota, Gabriela Stoppe, Geert Dom, Kirsten Catthoor, Eka Chkonia, Maria João Heitor Dos Santos, Diogo Telles, Peter Falkai, Philippe Courtet, Michal Patarák, Lubomira Izakova, Oleg Skugarevski, Stojan Barjaktarov, Dragan Babic, Goran Racetovic, Andrea Fiorillo, Bernardo Carpiniello, Maris Taube, Yuval Melamed, Jana Chihai, Doina Constanta Maria Cozman, Pavel Mohr, György Szekeres, Mirjana Delic, Ramunė Mazaliauskienė, Aleksandar Tomcuk, Nataliya Maruta, Philip Gorwood

**Affiliations:** 1 Department of Psychiatry and Psychological Medicine, University Hospital Centre Zagreb and School of Medicine, University of Zagreb, Zagreb, Croatia; 2 Department of Psychiatry and Psychotherapy, University Hospital, Ludwig-Maximilian University (LMU), Munich, Germany; 3 Mental Health Institute Hospital del Mar and Hospital del Mar Research Institute, Barcelona, CIBERSAM, Barcelona, Spain; 4 Universitat Pompeu Fabra, Barcelona, Spain; 5 Psychiatric University Clinic of Charité at St. Hedwig Hospital Berlin, Berlin, Germany; 6 Research Unit “Dr. Mirko Grmek”, Psychiatric Clinic “Sveti Ivan”, Zagreb, Croatia; 7 Department of Psychiatry, Helsinki University Central Hospital, Helsinki, Finland; 8 Royal College of Psychiatrists, London, UK; 9 Great Yarmouth Acute Service, Northgate Hospital/Norfolk & Suffolk NHS Foundation Trust, Great Yarmouth, UK; 10 Faculty of Psychology, University of Barcelona, Barcelona, Spain; 11 Department of Child and Adolescent Psychiatry, Institute of Psychiatry and Mental Health, Hospital General Universitario Gregorio Marañón, Instituto de Investigación Sanitaria Gregorio Marañón (IiSGM), CIBERSAM, ISCIII, School of Medicine, Universidad Complutense, Madrid, Spain; 12 Vrapče Psychiatric University Clinic, Zagreb, Croatia; 13 The National Mental Health Center, Baku, Azerbaijan; 14 Department of Adult Psychiatry, Mater Misericordiae University Hospital, Dublin, Ireland; 15 Department of Psychiatry and Addiction, Saint-Petersburg State University, Saint-Petersburg, Russia; 16 Department of Psychiatry, Hacettepe University Faculty of Medicine, Ankara, Turkey; 17 Affective Disorders Unit, Department of Psychiatry, Ege University Medicine Faculty, Izmir, Turkey; 18 Psychiatry and Department of Adult Psychiatry, Collegium Medicum Jagiellonian University, Cracow, Poland; 19 Pomeranian Medical University in Szczecin, Szczecin, Poland; 20 Clinic for Psychiatry, University of Kragujevac, Kragujevac, Serbia; 21 Swiss Society for Psychiatry and Psychotherapy, Switzerland; 22 MentAge – Counsil-Practice-Research, Basel, Switzerland; 23 Collaborative Antwerp Psychiatric Research Institute (CAPRI), University of Antwerp (UAntwerp), Antwerp, Belgium; 24 Department of Psychiatry, Tbilisi State Medical University, Tbilisi, Georgia; 25 Psychiatry and Mental Health Department, Hospital Beatriz Ângelo, Loures, Portugal; 26 Centro de Investigação Interdisciplinar em Saúde (CIIS), Universidade Católica Portuguesa, Lisbon, Portugal; 27 Faculdade de Medicina, Universidade Católica Portuguesa, Lisbon, Portugal; 28 Faculdade de Medicina, Universidade de Lisboa, Lisbon, Portugal; 29 Department of Emergency Psychiatry and Post Acute Care, Hôspital Lapeyronie, CHU Montpellier, Montpellier, France; 30 Department of Psychiatry, Slovak Medical University, Bratislava, Slovakia; 31 Roosevelt Teaching Hospital, Banská Bystrica, Slovakia; 32 Department of Psychiatry, Faculty of Medicine, Comenius University Bratislava, Bratislava, Slovakia; 33 Department of Psychiatry and Medical Psychology, Belarusian State Medical University, Minsk, Belarus; 34 University Clinic of Psychiatry, Faculty of Medicine, Ss. Cyril and Methodius University in Skopje, Skopje, Republic of North Macedonia; 35 Psychiatry Clinic, University Clinical Hospital Mostar, Mostar, Bosnia and Herzegovina; 36 Community Mental Health Center, Health Center Prijedor, Prijedor, Bosnia and Herzegovina; 37 Department of Psychiatry, University of Campania “L. Vanvitelli”, Naples, Italy; 38 University of Cagliari and Psychiatry Unit, Section of Psychiatry, Department of Medical Sciences and Public Health, University Hospital, Cagliari, Italy; 39 Department of Psychiatry and Narcology, Riga Stradiņš University, Riga Centre of Psychiatry and Narcology, Riga, Latvia; 40 Abarbanel Mental Health Center, Tel Aviv, Israel; 41 Department of Mental Health, Medical Psychology and Psychotherapy, Nicolae Testemitanu State University of Medicine and Pharmacy of the Republic of Moldova, Kishinev, Moldova; 42 Iuliu Hatieganu University of Medicine and Pharmacy, Cluj-Napoca, Romania; 43 Clinical Department, National Institute of Mental Health, Klecany, Czechia; 44 Third School of Medicine, Charles University, Prague, Czech Republic; 45 Semmelweis University, Budapest, Hungary; 46 University Psychiatric Clinic, Ljubljana, Slovenia; 47 Psychiatric Clinic, Lithuanian Health Sciences University Kaunas Hospital, Kaunas, Lithuania; 48 Special Psychiatric Hospital Kotor, Kotor, Montenegro; 49 Institute of Neurology, Psychiatry and Narcology, National Academy of Medical Sciences of Ukraine, Kharkiv, Ukraine; 50 Université Paris Cité, INSERM UMR1266, Paris, France; 51 CMME, GHU Paris Psychiatrie et Neurosciences, Hôpital Sainte-Anne, Paris, France

**Keywords:** Europe, guidelines, mental health, psychiatry, psychopharmacology, PTSD

## Abstract

**Background:**

Considering the recently growing number of potentially traumatic events in Europe, the European Psychiatric Association undertook a study to investigate clinicians’ treatment choices for post-traumatic stress disorder (PTSD).

**Methods:**

The case-based analysis included 611 participants, who correctly classified the vignette as a case of PTSD, from Central/ Eastern Europe (CEE) (*n* = 279), Southern Europe (SE) (*n* = 92), Northern Europe (NE) (*n* = 92), and Western Europe (WE) (*N* = 148).

**Results:**

About 82% woulduse antidepressants (sertraline being the most preferred one). Benzodiazepines and antipsychotics were significantly more frequently recommended by participants from CEE (33 and 4%, respectively), compared to participants from NE (11 and 0%) and SE (9% and 3%). About 52% of clinicians recommended trauma-focused cognitive behavior therapy and 35% psychoeducation, irrespective of their origin. In the latent class analysis, we identified four distinct “profiles” of clinicians. In Class 1 (*N* = 367), psychiatrists would less often recommend any antidepressants. In Class 2 (*N* = 51), clinicians would recommend trazodone and prolonged exposure therapy. In Class 3 (*N* = 65), they propose mirtazapine and eye movement desensitization reprocessing therapy. In Class 4 (*N* = 128), clinicians propose different types of medications and cognitive processing therapy. About 50.1% of participants in each region stated they do not adhere to recognized treatment guidelines.

**Conclusions:**

Clinicians’ decisions for PTSD are broadly similar among European psychiatrists, but regional differences suggest the need for more dialogue and education to harmonize practice across Europe and promote the use of guidelines.

## Introduction

Post-traumatic stress disorder (PTSD) and other trauma-related conditions are common and often occur comorbidly with other mental health conditions. According to surveys on prevalence using a randomly selected traumatic event, the lifetime prevalence of PTSD varies across the countries from 2 to 9%. They are therefore frequently found in everyday psychiatric clinical practice [1], given that there are many potentially life-threatening events [2]. Acute trauma without treatment may lead to maladaptive disorders and PTSD within days of the trauma [3]. The growing number of potentially traumatic recent events and stressors in Europe (i.e., war in Ukraine, COVID-19 pandemic, refugee waves) [4–6] means that an increase in trauma-related disorders is expected [7]. In a recent umbrella meta-analysis, psychological trauma has been identified as a transdiagnostic risk factor for several mental disorders [8]. According to the World Health Organization (WHO), it is estimated that in wars and disasters, the proportion of people with severe mental disorders increases by an average of 1% from a baseline of 2%–3%. In addition, the proportion of people with mild or moderate mental disorders, including most presentations of mood and anxiety disorders (including PTSD), may increase by 5%–10% from an estimated baseline of 10% [9]. Furthermore, chronic PTSD is associated with higher comorbidities of somatic conditions, including hypertension, hyperlipidemia, and metabolic syndrome, all of which can cause cardiovascular and cerebrovascular disease [10, 11].

On the European level, there is considerable variability of attitudes, procedures, and strategies in mental health care between clinicians and settings across different regions and countries. For example, we found significant differences in the predominant styles in decision-making, when comparing European regions and individual countries [12]. When it comes to service delivery, Northern and Western high-income countries have developed a large variety of multidisciplinary community-based services for people with mental health problems, with most patients being treated outside of psychiatric institutions [13], contrary to many Eastern European countries where psychiatric care is predominantly hospital-based. In addition, there is a significant overrepresentation of data from mental health services in Western and Northern European countries, due to a lack of data from Eastern Europe [14]. The latest National Institute for Health and Care Excellence (NICE) [15] guidelines from 2018 recommend mainly cognitive behavior therapy (CBT)-oriented trauma-focused interventions as first-line treatment for PTSD and, if pharmacological treatment is preferred by the client, venlafaxine or selective serotonin reuptake inhibitors (SSRIs), whereas the latest [16] International Society for Traumatic Stress Studies guidelines propose the following as first-line treatment for PTSD: trauma-focused CBT, cognitive processing therapy (CPT), cognitive therapy, eye movement desensitization and reprocessing (EMDR) therapy, and prolonged exposure (PE). First-line pharmacological treatment options include hereby SSRIs (fluoxetine, sertraline, paroxetine) or venlafaxine although trauma-focused psychological interventions are prioritized over pharmacotherapy. Burback et al. (2023) have comprehensively reviewed the current evidence for therapies in PTSD and proposed a staging model that may guide clinical treatment in the future [17].

Here, we were interested in investigating whether the sociocultural context of European regions is associated with differences in mental health care for trauma-related conditions and psychiatrists’ treatment choices in the treatment of PTSD.

Thus, this survey was conducted within the European Psychiatric Association (EPA) Ambassador Programme [12, 18] in order to identify therapeutic choices for treating PTSD among psychiatrists working in different European regions (Central and Eastern [CEE], Northern [NE], Southern [SE], and Western Europe [WE]).

## Methods

### Study design, setting, and participants

This study followed the design of previous EPA Ambassador Programme studies. The EPA Ambassadors Programme (www.europsy.net/advocacy/epa-ambassadors-programme) aims to establish a database of information on mental health practices and perspectives across Europe and different mental health disciplines. The program operates as a series of surveys on relevant topics aimed at all mental health professionals [12, 18]. We approached psychiatrists working in Europe, who were associated with the EPA community, including individual members of the EPA and its member associations, and attendees of the last 10 EPA congresses. In 2020, they were offered the opportunity to become “EPA Ambassadors” and to participate in EPA surveys. Initially, we sent an invitation email to previous EPA congress attendees, which are approximately 5,000 individuals. The EPA Council of National Psychiatric Associations, the Board, and the EPA Sections were then asked to distribute the invitations to their members. Responses were collected from April to December 2022 using an online questionnaire. The study was open to psychiatrists and psychiatry specialist trainees working in Europe. The authors declare that all procedures contributing to this work complied with the ethical standards of the relevant national and institutional committees for human experimentation and with the Helsinki Declaration of 1975, as revised in 2008 and 2013 [19]. The study was approved by the Ethical Committee of the Zagreb University Hospital Centre (number 02/1013AG).

### Variables

Treatment attitudes were assessed using vignettes describing two typical cases of PTSD. The two vignettes differed only in the type of trauma, that is war trauma (case 1) versus civil trauma (case 2) and the country of origin of the patient. The cases were developed by experts in the field of psychotraumatology and psychiatry (authors FP, BA, MSH, PG, MRK) on the basis of their consensus. The cases were piloted with 20 members of the EPA before the survey was launched, and the survey was subsequently revised according to their feedback.

As we wish to focus on PTSD rather than complex PTSD (cPTSD), which will be a new diagnostic category in ICD-11, we only present results based on the clinical vignette of a typical civilian trauma, rather than the clinical vignette of war trauma, which represents recurrent or continuous traumatic events that are associated with a higher risk of cPTSD. Responses offered multiple choices for diagnoses, assessment tools, and treatment approaches balancing pharmacological and non-pharmacological treatment options. “A 34-year-old woman from the main city of your country has arrived at your place of work after she began to experience depression, insomnia, and “flashbacks” of her experience of an armed robbery where her colleague was killed at her place at the bank where she works. Very soon, she became particularly concerned with watching TV news reporting of the event, and the more she watched news, the more she became agitated about it and remembered about the coworkers who talked about the event. She became anxious, emotionally overwhelmed, and finally emotionally distant from her children.” Recorded were responses on sociodemographic data (age, gender), the nature of clinicians’ expertise, training, and practice (time since qualifying as consultant psychiatrists, subspecialty, work position, type of practice, and clinical setting), as well as place of work (including specifically, use of clinical guidelines in clinical practice and availability of treatment options for PTSD).

The final questionnaire is available as (Supplementary Material.

### Statistical analysis

We used a post-stratification of nonresponse weighted at the country level to correct for imbalanced response patterns across different European regions as a source of potential bias in our survey. We calculated these by dividing the proportion of each country’s population in the total European population by the proportion of each country’s sample in the total sample (Supplementary Figure 1). We categorized the countries into four regions according to EuroVoc (Supplementary Table 1): CEE, NE, WE, and SE. We used latent class analysis (LCA) to classify participants into distinct latent groups based on their responses to various treatment choices and characteristics. The optimal number of latent classes was determined based on several goodness-of-fit indices, including log-likelihood, number of estimated parameters (np), consistent Akaike information criterion (CAIC), Bayesian information criterion (BIC), and sample size adjusted BIC (SABIC). The model with the lowest values of CAIC, BIC and SABIC, and a significant LRT p-value, was considered the best fit model. Once the optimal number of latent classes had been identified, participants were assigned to the class for which they had the highest posterior probability. Descriptive statistics were then used to characterize each latent class in terms of demographic variables, professional characteristics, and treatment choices. Differences in categorical variables between latent classes were tested for statistical significance using chi-squared tests, whereas continuous variables were compared using ANOVA. All analyses related to the LCA were conducted using Stata’s gsem command, and the results were interpreted in the context of the broader study objectives. We corrected the statistical significance for multiple testing using the Benjamini–Hochberg procedure with a false discovery rate (FDR) set at <5%. Analyses were performed using StataCorp. 2019. *Stata Statistical Software, Release 16.* College Station, TX, USA: StataCorp LLC. The manuscript was written according to the STROBE guidelines for reporting cross-sectional studies [20].

## Results

### Participant characteristics

The online survey was completed by 1105 mental health professionals worldwide. However, as this analysis focused on Europe only, the final sample consisted of 948 participants, including 835 (88.1%) psychiatrists and 113 (11.9%) psychiatry specialist trainees working in 39 European countries. The regional distribution of the final sample notably deviated from the regional distribution of the target population of European psychiatrists (Supplementary Table 1 and Supplementary Figure 1). As response rates were unevenly distributed across countries, we corrected for these imbalances using post-stratification weighting. There were 139 (14.7%) missing data for the civil PTSD vignette. As imputation would not have been valid, we conducted further analysis on 809 respondents with complete data. In the final sample, only European psychiatrists and psychiatry residents who responded to Case 2 and identified PTSD as the most likely diagnosis were included (Supplementary Table 2). The sample consisted of 611 participants from the four European regions: CEE (*n* = 279), SE (*n* = 92), NE (*n* = 92), and WE (*n* = 148) (Table 1). We found almost no differences between respondents who correctly identified PTSD and those who did not in most of the variables examined, including age, gender, work setting, and experience in traumatology or psychotherapy, nor in self-reports regarding adherence to clinical guidelines (Supplementary Table 3). In the final sample, the majority (72.3%) used a scale/instrument to validate the diagnosis or measure the severity of symptoms and treatment efficacy, with no significant differences between regions.

**Table 1.** Description of participants; raw, unweighted data; total sample (*n* = 611)

	CE Europe (*n* = 279)	Southern (*n* = 92)	Northern (*n* = 92)	Western (*n* = 148)	Total (*n* = 611)
Age (years), mean (SD)	44 (11)	43 (13)	49 (12)	51 (12)	46 (12)
Gender					
Men	106 (38.0)	48 (52.2)	34 (37.0)	81 (54.7)	269 (44.0)
Women	173 (62.0)	44 (47.8)	58 (63.0)	67 (45.3)	342 (56.0)
Professional status					
Residents	41 (14.7)	14 (15.2)	6 (6.5)	11 (7.4)	72 (11.8)
Psychiatrists	238 (85.3)	78 (84.8)	86 (93.5)	137 (92.6)	539 (88.2)
Workplace					
University department	139 (49.8)	34 (37.0)	26 (28.3)	37 (25.0)	236 (38.6)
Public hospital	106 (38.0)	51 (55.4)	26 (28.3)	62 (41.9)	245 (40.1)
Private hospital or private practice	68 (24.4)	21 (22.8)	32 (34.8)	32 (21.6)	153 (25.0)
Community mental health	18 (6.5)	7 (7.6)	5 (5.4)	33 (22.3)	63 (10.3)
Work experience (years), mean (SD)	16 (11)	15 (12)	19 (10)	21 (12)	18 (11)
Certified psychotherapists	108 (38.7)	46 (50.0)	37 (40.2)	67 (45.3)	258 (42.2)
Certified psychotherapists’ main area					
Cognitive behavioral therapy	54 (49.1)	17 (37.8)	7 (21.2)	20 (30.8)	98 (38.7)
Psychodynamic oriented	10 (9.1)	9 (20.0)	3 (9.1)	13 (20.0)	35 (13.8)
Integrative psychotherapy	11 (10.0)	4 (8.9)	7 (21.2)	12 (18.5)	34 (13.4)
Systemic family therapy	11 (10.0)	3 (6.7)	4 (12.1)	7 (10.8)	25 (9.9)
Psychoanalytic psychotherapy	2 (1.8)	4 (8.9)	6 (18.2)	4 (6.2)	16 (6.3)
EMDR	4 (3.6)	3 (6.7)	3 (9.1)	4 (6.2)	14 (5.5)
Other	18 (16.4)	5 (11.1)	3 (9.1)	5 (7.7)	31 (12.3)
Psychotraumatologists	52 (18.6)	11 (12.0)	8 (8.7)	36 (24.3)	107 (17.5)
PTSD patients monthly, mean (SD)	6 (9)	7 (14)	5 (9)	10 (13)	7 (11)
Availability of trauma-focused therapies					
Trauma-focused CBT	116 (41.6)	38 (41.3)	44 (47.8)	84 (56.8)	282 (46.2)
EMDR	44 (15.8)	23 (25.0)	39 (42.4)	84 (56.8)	190 (31.1)
Psychodynamic oriented	75 (26.9)	23 (25.0)	32 (34.8)	46 (31.1)	176 (28.8)
Cognitive processing therapy	70 (25.1)	19 (20.7)	21 (22.8)	22 (14.9)	132 (21.6)
Narrative exposure therapy	18 (6.5)	13 (14.1)	4 (4.3)	26 (17.6)	61 (10.0)
Prolonged exposure	19 (6.8)	3 (3.3)	11 (12.0)	19 (12.8)	52 (8.5)
Other trauma-focused psychotherapy	51 (18.3)	4 (4.3)	24 (26.1)	46 (31.1)	125 (20.5)
No trauma-focused therapy	73 (26.2)	23 (25.0)	22 (23.9)	24 (16.2)	142 (23.2)
Guidelines followed					
National guidelines	72 (25.8)	12 (13.0)	41 (44.6)	41 (27.7)	166 (27.2)
NICE	68 (24.4)	24 (26.1)	13 (14.1)	56 (37.8)	161 (26.4)
WHO	70 (25.1)	12 (13.0)	5 (5.4)	16 (10.8)	103 (16.9)
APA	36 (12.9)	17 (18.5)	5 (5.4)	16 (10.8)	74 (12.1)
ISTSS	7 (2.5)	4 (4.3)	7 (7.6)	10 (6.8)	28 (4.6)
U.S. Department of Defense	8 (2.9)	4 (4.3)	0 (0.0)	1 (0.7)	13 (2.1)
No specific guidelines	137 (49.1)	58 (63.0)	44 (47.8)	67 (45.3)	306 (50.1)

Abbreviations: CBT, cognitive behavioral therapy; CE, central and eastern; EMDR, eye movement desensitization and reprocessing; PTSD, post-traumatic stress disorder; SD, standard deviation.

*Note*: Data are presented as number (percentage) of participants if not stated otherwise.

### Clinicians’ treatment choices

The majority of participants would use antidepressants (82%), but we observed statistically significant regional differences. Participants from NE and WE were less likely to recommend antidepressants: 58 and 77%, respectively, compared to participants from CEE (89%) and SE (92%), respectively (Tables 2 and 3). The odds ratio for the use of any antidepressant in SE versus NE was 7.88 (95% CI 2.61; 23.75; *p* < 0.001; FDR < 5%). There were also significant regional differences in the specific choice of antidepressant. Venlafaxine would be preferred by participants from the CEE region compared to the NE and WE regions, sertraline would be preferred by participants from the SE region compared to the CEE, NE, and WE regions, and trazodone would be the least popular choice of participants from the NE region compared to all three regions. Significant differences in preferred medications were found, especially for concomitant medications. Benzodiazepines/sedatives were preferred in CEE compared to NE and WE, and in SE compared to NE (Tables 2 and 3). Antipsychotics were preferred in CEE regions versus NE regions (Tables 2 and 3). In terms of non-pharmacological methods, the majority of participants would recommend trauma-focused CBT (52%) and psychoeducation (35%), with no significant differences between regions (Tables 2 and 3). However, there were significant differences between regions for other specific psychotherapies. CBT and cognitive processing would be recommended slightly more often in CEE compared to NE and WE and in SE compared to WE. Systemic therapy would be recommended more often in CEE compared to SE and PE therapy in CEE compared to NE. Interestingly, other psychotherapies would be recommended more often in WE than in the other three regions (Tables 2 and 3).

**Table 2.** Treatment choices; weighted data (*n* = 611)

	CE Europe (*n* = 279)	Southern (*n* = 92)	Northern (*n* = 92)	Western (*n* = 148)	Total (*n* = 611)
Pharmacotherapy					
Any antidepressant	89 (82–94)	92 (82–96)	58 (41–73)	77 (66–85)	82 (76–86)
Particular antidepressants					
Sertraline	63 (53–72)	80 (69–88)	49 (33–65)	61 (49–71)	64 (58–70)
Venlafaxine	17 (11–25)	11 (5–23)	5 (3–8)	7 (3–12)	10 (7–14)
Other SSRI	36 (27–46)	21 (12–33)	9 (6–13)	15 (9–22)	21 (17–25)
Mirtazapine	9 (6–13)	17 (9–31)	17 (9–31)	14 (8–23)	14 (10–19)
Trazodone	13 (8–20)	13 (7–22)	5 (3–9)	11 (7–16)	11 (9–15)
Tricyclic antidepressants	1 (0–5)	1 (0–4)	1 (0–3)	3 (0–13)	2 (1–6)
Other drugs					
Benzodiazepines	33 (24–42)	23 (14–37)	11 (4–27)	9 (7–11)	18 (15–21)
Anticonvulsants	3 (1–7)	3 (0–18)	0 (0–0)	0 (0–0)	1 (1–3)
Antipsychotics	4 (2–7)	2 (1–6)	0 (0–0)	3 (1–13)	3 (1–6)
Psychotherapy					
Trauma-focused CBT	46 (36–56)	43 (32–55)	63 (42–80)	57 (46–67)	52 (45–58)
Psychoeducation about trauma	31 (23–41)	24 (15–37)	40 (23–61)	41 (30–52)	35 (29–42)
Cognitive behavioral therapy	42 (32–52)	30 (19–45)	13 (9–17)	18 (12–28)	26 (21–32)
Cognitive processing therapy	33 (24–42)	23 (14–37)	11 (4–27)	9 (7–11)	18 (15–21)
EMDR	9 (6–13)	17 (9–31)	17 (9–31)	14 (8–23)	14 (10–19)
Prolonged exposure	13 (8–20)	13 (7–22)	5 (3–9)	11 (7–16)	11 (9–15)
Psychodynamic psychotherapy	9 (5–17)	7 (3–17)	5 (3–10)	11 (6–19)	9 (6–14)
Narrative exposure therapy	1 (0–5)	1 (0–4)	1 (0–3)	3 (0–13)	2 (1–6)
Systemic therapy	4 (1–11)	0 (0–0)	0 (0–2)	0 (0–0)	1 (0–3)
Other trauma-focused psychotherapy	3 (1–7)	3 (0–18)	0 (0–0)	0 (0–0)	1 (1–3)
Other therapy	2 (1–3)	1 (0–4)	1 (0–3)	12 (6–22)	7 (4–12)

Abbreviations: CBT, cognitive behavioral therapy; CE, central and eastern; EMDR, eye movement desensitization and reprocessing.

*Note*: Data are presented as weighted percentage (95% confidence interval).

**Table 3.** Regional differences in preferred treatment; weighted data (*n* = 611)

	CEE-SE	CEE-NE	CEE-WE	SE-NE	SE-WE	NE-WE
Pharmacotherapy						
Any antidepressant	0.77 (0.26–2.29)	6.10 (2.41–15.42)^a^	2.57 (1.13–5.86)^b^	7.88 (2.61–23.75)^a^	3.32 (1.20–9.17)^b^	0.42 (0.18–0.98)
Particular antidepressants						
Venlafaxine	1.64 (0.60–4.50)	3.75 (1.84–7.63)^a^	2.78 (1.18–6.56)^b^	2.28 (0.84–6.18)	1.69 (0.56–5.11)	0.74 (0.32–1.72)
Sertraline	0.43 (0.21–0.88)^b^	1.82 (0.84–3.94)	1.13 (0.62–2.08)	4.19 (1.75–10.02)^c^	2.61 (1.26–5.38)^b^	0.62 (0.28–1.38)
Other SSRI	2.12 (1.01–4.46)	5.47 (3.09–9.66)^a^	3.24 (1.69–6.21)^a^	2.58 (1.24–5.38)^b^	1.53 (0.69–3.40)	0.59 (0.31–1.12)
Trazodone	1.00 (0.44–2.25)	2.91 (1.28–6.63)^b^	1.24 (0.62–2.48)	2.91 (1.17–7.26)^b^	1.24 (0.56–2.75)	0.43 (0.19–0.96)^b^
Mirtazapine	0.48 (0.21–1.13)	0.49 (0.20–1.19)	0.64 (0.30–1.35)	1.02 (0.35–2.98)	1.32 (0.50–3.46)	1.29 (0.48–3.49)
Tricyclic antidepressants	1.21 (0.15–9.74)	1.60 (0.22–11.69)	0.46 (0.05–4.31)	1.32 (0.17–10.15)	0.38 (0.04–3.72)	0.28 (0.03–2.57)
Other drugs						
Benzodiazepines	1.60 (0.74–3.44)	3.83 (1.21–12.13)^b^	4.92 (3.06–7.91)^a^	2.40 (0.68–8.41)	3.08 (1.57–6.05)^c^	1.28 (0.43–3.84)
Anticonvulsants	1.04 (0.13–8.63)	–	–	–	–	–
Antipsychotics	1.84 (0.51–6.68)	12.64 (1.58–101.11)^b^	1.31 (0.23–7.38)	6.85 (0.72–64.74)	0.71 (0.10–4.84)	0.10 (0.01–1.29)
Psychotherapy						
Trauma-focused CBT	1.10 (0.60–2.03)	0.50 (0.20–1.26)	0.64 (0.35–1.16)	0.46 (0.18–1.18)	0.58 (0.31–1.10)	1.28 (0.50–3.27)
Psychoeducation about trauma	1.41 (0.68–2.95)	0.67 (0.27–1.68)	0.67 (0.36–1.23)	0.48 (0.17–1.31)	0.47 (0.22–0.99)	0.99 (0.39–2.49)
Cognitive behavioral therapy	1.67 (0.80–3.51)	5.00 (2.94–8.48)^a^	3.16 (1.63–6.12)^c^	2.99 (1.47–6.07)^c^	1.89 (0.84–4.25)	0.63 (0.34–1.18)
Cognitive processing therapy	1.60 (0.74–3.44)	3.83 (1.21–12.13)^b^	4.92 (3.06–7.91)^a^	2.40 (0.68–8.41)	3.08 (1.57–6.05)^c^	1.28 (0.43–3.84)
EMDR	0.48 (0.21–1.13)	0.49 (0.20–1.19)	0.64 (0.30–1.35)	1.02 (0.35–2.98)	1.32 (0.50–3.46)	1.29 (0.48–3.49)
Prolonged exposure	1.00 (0.44–2.25)	2.91 (1.28–6.63)^b^	1.24 (0.62–2.48)	2.91 (1.17–7.26)^b^	1.25 (0.56–2.75)	0.43 (0.19–0.96)
Psychodynamic psychotherapy	1.43 (0.42–4.82)	1.84 (0.74–4.59)	0.86 (0.33–2.26)	1.28 (0.39–4.21)	0.60 (0.18–2.05)	0.47 (0.19–1.18)
Narrative exposure therapy	1.21 (0.15–9.74)	1.60 (0.22–11.69)	0.46 (0.05–4.31)	1.32 (0.17–10.15)	0.38 (0.04–3.72)	0.28 (0.03–2.57)
Systemic therapy	15.25 (1.68–138.30)^b^	14.11 (1.51–131.74)^b^	–	0.93 (0.06–14.60)	–	–
Other trauma-focused psychotherapy	1.04 (0.13–8.63)	–	–	–	–	–
Other therapy	1.38 (0.34–5.62)	1.44 (0.45–4.62)	0.13 (0.05–034)^a^	1.05 (0.22–5.07)	0.09 (0.02–0.39)^c^	0.09 (0.03–0.30)^a^

Abbreviations: CBT, cognitive behavioral therapy; CEE, central and eastern Europe; EMDR, eye movement desensitization and reprocessing – statistic could not be calculated; NE, Northern Europe; SE, Southern Europe; SSRI, selective serotonin reuptake inhibitors; WE, Western Europe.

*Note*: Data are presented as weighted odds ratio (95% confidence interval). In each pair, a second region is the referent (odds = 1.00).

a Statistically significant at *p* < 0.001 with a false discovery rate <5%.

b Statistically significant at *p* < 0.05 with a false discovery rate <5%.

c Statistically significant at *p* < 0.01 with a false discovery rate <5%.

### Latent classes based on clinicians’ treatment choices

The model with four latent classes demonstrated the best fit to the empirical data (Supplementary Table 4). The number of participants classified into each of the four latent classes according to the highest posterior probability was 367 (60.1%) in Class 1 (“Conventional Antidepressant”), 51 (8.4%) in Class 2 (“Trazodone and Exposure Therapy”), 65 (10.6%) in Class 3 (“Mirtazapine and EMDR”), and 128 (21.0%) in Class 4 (“Comprehensive medication and CPT”).

Any antidepressant (vs. no antidepressant) would be recommended less often by respondents in the first latent class (Table 4). In the other three latent classes, all or almost all respondents would use an antidepressant. Psychiatrists in the first latent class were most likely to recommend sertraline, as were psychiatrists in the second and fourth latent classes. All psychiatrists in the second latent class would recommend trazodone and PE therapy. The third latent class was characterized by the use of mirtazapine and EMDR therapy, and the fourth latent class was characterized by the use of a large number of different antidepressants, significant use of benzodiazepines, anticonvulsants, and CPT.

**Table 4.** Clinicians’ treatment choices in different latent classes

	Class 1 (*n* = 367)	Class 2 (*n* = 51)	Class 3 (*n* = 65)	Class 4 (*n* = 128)	p
Pharmacotherapy					
Any antidepressants	269 (73.3)	51 (100.0)	65 (100.0)	125 (97.7)	<0.001
Particular antidepressants					
Venlafaxine	45 (12.3)	10 (19.6)	10 (15.4)	27 (21.1)	0.081
Sertraline	211 (57.5)	31 (60.8)	30 (46.2)	77 (60.2)	0.264
Other SSRI	90 (24.5)	20 (39.2)	15 (23.1)	53 (41.4)	0.001
Trazodone	0 (0.0)	51 (100.0)	0 (0.0)	22 (17.2)	<0.001
Mirtazapine	0 (0.0)	15 (29.4)	65 (100.0)	32 (25.0)	<0.001
Tricyclic antidepressants	3 (0.8)	3 (5.9)	2 (3.1)	3 (2.3)	0.054
Other drugs					
Benzodiazepines	0 (0.0)	0 (0.0)	0 (0.0)	128 (100.0)	<0.001
Anticonvulsants	3 (0.8)	1 (2.0)	3 (4.6)	7 (5.5)	0.012
Antipsychotics	16 (4.4)	2 (3.9)	1 (1.5)	11 (8.6)	0.130
Psychotherapy					
Trauma-focused CBT	179 (48.8)	28 (54.9)	36 (55.4)	54 (42.2)	0.249
Psychoeducation about trauma	125 (34.1)	22 (43.1)	22 (33.8)	51 (39.8)	0.444
Cognitive behavioral therapy	110 (30.0)	21 (41.2)	24 (36.9)	47 (36.7)	0.230
Cognitive processing therapy	0 (0.0)	0 (0.0)	0 (0.0)	128 (100.0)	<0.001
EMDR	0 (0.0)	15 (29.4)	65 (100.0)	32 (25.0)	<0.001
Prolonged exposure	0 (0.0)	51 (100.0)	0 (0.0)	22 (17.2)	<0.001
Psychodynamic psychotherapy	34 (9.3)	4 (7.8)	7 (10.8)	10 (7.8)	0.900
Narrative exposure therapy	3 (0.8)	3 (5.9)	2 (3.1)	3 (2.3)	0.054
Systemic therapy	3 (0.8)	1 (2.0)	1 (1.5)	9 (7.0)	0.001
Other trauma-focused psychotherapy	3 (0.8)	1 (2.0)	3 (4.6)	7 (5.5)	0.012
Other therapy	23 (6.3)	3 (5.9)	5 (7.7)	9 (7.0)	0.965

*Note*: Class 1: conventional antidepressant; Class 2: trazodone and exposure therapy; Class 3: mirtazapine and EMDR; Class 4: comprehensive medication and CPT.

Significant differences were observed across the latent class regional distribution (Table 5). The age of the clinician also varied significantly across classes, with Class “Comprehensive medication and CPT” having the lowest mean age of 43 years. Differences in work experience were also evident, with Class “Mirtazapine and EMDR” having the highest mean work experience of 21 years. The availability of trauma-focused therapies like EMDR was significantly higher in Class 3 patients (49.2%).

**Table 5.** Description of the latent classes

	Class 1 (*n* = 367)	Class 2 (*n* = 51)	Class 3 (*n* = 65)	Class 4 (*n* = 128)	p
Region					
CE Europe	143 (39.0)	19 (37.3)	23 (35.4)	94 (73.4)	<0.001
Southern Europe	52 (14.2)	16 (31.4)	9 (13.8)	15 (11.7)	
Northern Europe	49 (16.1)	7 (13.7)	16 (24.6)	10 (7.8)	
Western Europe	47 (12.0)	9 (17.6)	17 (26.2)	9 (7.0)	
Age (years), mean (SD)	47 (12.2)	47 (13.8)	50 (12.7)	43 (11.0)	0.001
Gender					
Men	159 (43.3)	27 (52.9)	28 (43.1)	55 (43.0)	0.615
Women	208 (56.7)	24 (47.1)	37 (56.9)	73 (57.0)	
Professional status					
Residents	42 (11.4)	4 (7.8)	7 (10.8)	19 (14.8)	0.568
Psychiatrists	325 (88.6)	47 (92.2)	58 (89.2)	109 (85.2)	
Workplace					
University department	131 (35.7)	20 (39.2)	27 (41.5)	58 (45.3)	0.263
Public hospital	140 (38.1)	23 (45.1)	24 (36.9)	58 (45.3)	0.418
Private hospital or private practice	96 (26.2)	16 (31.4)	16 (24.6)	25 (19.5)	0.333
Community mental health	44 (12.0)	2 (3.9)	7 (10.8)	10 (7.8)	0.236
Work experience (years), mean (SD)	18 (11)	18 (12)	21 (13)	15 (10)	0.002
Certified psychotherapist	171 (46.6)	14 (27.5)	29 (44.6)	44 (34.4)	0.013
Certified psychotherapists’ main area					
Cognitive behavioral therapy	68 (40.7)	5 (29.4)	10 (34.5)	15 (37.5)	0.143
Psychodynamic oriented	21 (12.6)	4 (23.5)	5 (17.2)	5 (12.5)	
Integrative psychotherapy	21 (12.6)	1 (5.9)	7 (24.1)	5 (12.5)	
Systemic family therapy	15 (9.0)	1 (5.9)	1 (3.4)	8 (20.0)	
Psychoanalytic psychotherapy	10 (6.0)	4 (23.5)	2 (6.9)	0 (0.0)	
EMDR	10 (6.0)	1 (5.9)	2 (6.9)	1 (2.5)	
Other	22 (13.2)	1 (5.9)	2 (6.9)	6 (15.0)	
Psychotraumatologists	63 (17.2)	6 (11.8)	13 (20.0)	25 (19.5)	0.607
PTSD patients monthly, mean (SD)	8 (13)	5 (6)	8 (9)	5 (7)	0.094
Availability of trauma-focused therapies					
Trauma-focused CBT	172 (46.9)	21 (41.2)	38 (58.5)	51 (39.8)	0.086
EMDR	124 (33.8)	9 (17.6)	32 (49.2)	25 (19.5)	<0.001
Psychodynamic oriented	104 (28.3)	14 (27.5)	23 (35.4)	35 (27.3)	0.662
Cognitive processing therapy	74 (20.2)	10 (19.6)	11 (16.9)	37 (28.9)	0.142
Narrative exposure therapy	34 (9.3)	5 (9.8)	7 (10.8)	15 (11.7)	0.876
Prolonged exposure	35 (9.5)	1 (2.0)	8 (12.3)	8 (6.3)	0.148
Other trauma-focused psychotherapy	71 (19.3)	8 (15.7)	17 (26.2)	29 (22.7)	0.446
No trauma-focused therapy	83 (22.6)	13 (25.5)	10 (15.4)	36 (28.1)	0.242
Guidelines followed					
National guidelines	106 (28.9)	11 (21.6)	16 (24.6)	33 (25.8)	0.639
NICE	96 (26.2)	10 (19.6)	25 (38.5)	30 (23.4)	0.083
WHO	54 (14.7)	8 (15.7)	9 (13.8)	32 (25.0)	0.052
APA	38 (10.4)	7 (13.7)	10 (15.4)	19 (14.8)	0.433
ISTSS	17 (4.6)	4 (7.8)	4 (6.2)	3 (2.3)	0.380
U.S. Department of Defense	8 (2.2)	1 (2.0)	2 (3.1)	2 (1.6)	0.921
No specific guidelines	184 (50.1)	30 (58.8)	30 (46.2)	62 (48.4)	0.552

Abbreviations: CBT, cognitive behavioral therapy;; CE, central and eastern; EMDR, eye movement desensitization and reprocessing; PTSD, post-traumatic stress disorder; SD, standard deviation; V, Cramer’s coefficient of concordance.

*Note*: Data are presented as number (percentage) of participants if not stated otherwise. Class 1: conventional antidepressant; Class 2: trazodone and exposure therapy; Class 3: mirtazapine and EMDR; Class 4: comprehensive medication and CPT.

## Discussion

In this study, we investigated clinicians’ therapeutic choices for treating PTSD among psychiatrists and psychiatry specialist trainees working in mental health services in four European regions. Notably, almost 80% of the participants correctly identified the diagnosis described in the case vignette, regardless of the European region in which they worked. However, among those who correctly identified PTSD, preferred treatment choices often differed across European regions.

### Choice of pharmacological treatment

When comparing pharmacological interventions, we found that preferences for antidepressants in PTSD were similar across European regions, with around 80% of the participants recommending antidepressants, although significantly more so in SE and CEE regions (around 90%) compared to NE and WE. However, we found significant differences when comparing the preferred specific medications across European regions. While sertraline was the preferred first choice in all regions, it was largely predominant in SE. The second preferred antidepressant was “other SSRI,” but at different rates compared to other choices across regions. The third preferred antidepressant differed between regions, with mirtazapine somewhat predominant in NE and WE, venlafaxine in CEE, and trazodone least popular in NE.

The largest difference in medication choices was found for benzodiazepines and antipsychotics: benzodiazepines would be recommended by about a third of participants from CEE and by about a fifth of participants from SE, compared to less than 10% of participants from WE and NE. The high prescription rate of benzodiazepines in psychiatry and general medicine worldwide is often criticized because of concerns regarding addiction, intoxication, and mortality and therefore requires consideration [21]. However, regarding the particular importance of benzodiazepines in CEE and SE regions, it is possible that this is due to different clinical presentations of PTSD, that is, with more cPTSD characteristics, as countries from these regions have been devastated by wars in recent decades [22]. Some studies have suggested that use is more widespread in Western countries [23], but there may be a lack of data from CEE countries, as some authors have pointed out [14]. However, these results are consistent with studies from Eastern Europe indicating a high number of patients treated with benzodiazepines for long periods of time [24]. Interestingly, antipsychotics were more likely to be recommended among participants in CEE compared to NE countries. As with benzodiazepines, this may be due to the complex presentation of PTSD in patients from the CEE and SE regions, as countries from these regions have been devastated by war in recent decades.

When it comes to the specific choice of medication, the availability of some medications may explain the choice, for example, mirtazapine may not be as readily available in some CEE countries as, for example, trazodone. However, these results also appear to be related to a complex interplay of historical, cultural, and socioeconomic aspects that show differences between the four European regions and have subsequently contributed to differences in the training of mental health professionals and the organization of mental health services. For example, the educational and organizational characteristics of mental health services in CEE countries have been largely similar for decades in most of the Commonwealth of Independent States, which includes the largest part of the former Soviet Union’s member states [14], and may still influence clinicians’ decision-making processes 12], regardless of the recent changes in European mental health systems. On the other hand, NE and WE high-income countries have introduced a wide range of multidisciplinary community-based services for people with mental health problems and reorganized mental health care services toward recovery-oriented care models and a move toward human rights, social inclusion, and empowerment in recent decades [25], which may also have influenced the decreased use of medication in favor of non-pharmacological methods. These changes may have ultimately led to a shift from medication to psychological interventions. Indeed, we identified four distinct “profiles” of clinicians using LCA, some of which were associated with European regions, possibly indicating a different clinical training background, as described above: clinicians from Class “Conventional Antidepressant”(the most predominant one, associated with the highest percentage of psychotherapists with Class “Mirtazapine and EMDR”) would use antidepressants, usually sertraline, less often than other classes; clinicians from Class “Trazodone and Exposure Therapy” (associated with the SE region) would recommend trazodone and PE psychotherapy; clinicians from Class “Mirtazapine and EMDR” (associated with older age and more experience, status as a certified psychotherapist, availability of EMDR therapy, and WE and NE regions) would recommend mirtazapine and EMDR; and clinicians from Class “Comprehensive medication and CPT” (associated with the CE region) would recommend various antidepressants, benzodiazepines, and CPT.

### Choice of non-pharmacological treatment

Approximately 40% of the participants, with no significant differences between the four regions, reported that they were certified psychotherapists. In the LCA, the status of certified psychotherapist contributed to the affinity to Class “Conventional Antidepressant” and Class “Mirtazapine and EMDR” “profiles” of clinicians based on their choice of treatment for PTSD. Of all certified psychotherapists (42%), the vast majority were trained in CBT (39%), especially in the CEE countries. The number seems high [26], especially for regions other than NE or WE, because in most European countries outside these regions, becoming a licensed psychotherapist requires additional self-funded training on top of the regular education programs in psychiatry. In some of the WE and NE countries, psychotherapy training (mainly for CBT and in some countries also for systemic therapy, psychodynamic, and integrative psychotherapy) is offered and fully paid for within the regular psychiatry training program [26]. In line with this, and with the reported availability of psychotherapy services at their place of work, about 40% of respondents would also recommend non-pharmacological methods for PTSD – psychoeducation and trauma-focused CBT, with no regional differences. When comparing regions, it seems that some therapies (i.e., CPT, PE therapy) are more popular in CEE especially compared to NE regions, while some other psychotherapies are more popular in WE. According to our results, this distribution is not related to psychotherapeutic the training of the participants, but rather to the availability of the specific psychotherapeutic services at their place of work. It is possible that availability may explain some of the similarities between NE and WE in the treatment of PTSD – primarily in the use of similar psychotherapeutic approaches. While these findings are likely to be related to a complex interplay of general views on mental health, prescription practices, availability of resources, and further socioeconomic factors shaping mental health care in European regions, some of these aspects are certainly related to government policies and financial support for the development of mental health services within single countries. For example, the use of EMDR, as one of the newer evidence-based therapies for PTSD, has been introduced into psychiatric practice more recently than trauma-focused CBT or cognitive processing and may not be widely available in CEE countries [27]. Concordantly, in the LCA, where we identified four distinct “profiles “of clinicians in the sample based on their choice of treatment for PTSD, the availability of EMDR was one of the factors significantly associated with Class “Mirtazapine and EMDR” of participants. Psychiatric societies in NE and WE countries may have a stronger educational/scientific reputation in their countries and have more influence on government policies than in CEE countries, where this process may still be developing.

### The use of clinical guidelines

While the educational impact (as well as the influence on government policy) is also achieved by using clinical guidelines, it is interesting to note that about half of the participants reported that they do not use guidelines in clinical practice, regardless of the region of work. Of those who use clinical guidelines, participants from NE and WE seem to prefer national guidelines (WE also prefer NICE guidelines, which can be considered national at least for UK participants). Participants from SE and CEE would prefer national, NICE, and WHO guidelines (and APA in SE countries). This, again, supports the above-mentioned assumption that compared to SE and CEE, psychiatric societies in NE and WE countries have a stronger educational/scientific reputation among their colleagues and among their governments. Indeed, in the LCA, age and work experience were highest in Class “Mirtazapine and EMDR” of participants, which was predominantly found in NE and WE countries.

However, as about half of the participants stated that they did not adhere to the guidelines, and none of the named guidelines recommended regular concomitant use of benzodiazepines or mood stabilizers, we can also assume that other factors, such as general views, customs, available resources, and unknown factors, may contribute to specific regional treatment choices. In particular, given the historical background and nature of the political systems in the CEE until the 1990s, it is possible that most clinical knowledge was acquired through clinical experience and practice passed on to younger generations by their mentors, especially considering the publication gaps in these countries [14].

### Limitations of the study

The study had several limitations that have been described elsewhere [12]. Briefly, we cannot give clear estimation of the response rate nor claim that the sample is fully representative at the national level because of low response rates in individual countries and a possible association of nonresponse with specific preferred treatment approaches. The overall number of participants was comprised a small proportion of all psychiatrists, especially in some countries. We used EuroVoc to categorize countries into regions, which could potentially lead to the grouping of countries with different characteristics [28]. In addition, this study used a non-standardized tool to measure treatment attitudes based on clinical cases. Although the case vignettes were developed using consensus agreement with the international experts in the field of psychotraumatology, they were not designed to differentially assess diagnostic precision or differential diagnostic competencies in the spectrum of trauma-related disorders, or even validated nor were differences in culture and organization of healthcare in the participating countries considered when creating the survey. Thus, our survey represents a first approximation to this topic, but does not assess the level of clinical knowledge, competencies, and prejudices in the field of trauma-related disorders or include current developments, that is, the novel field of complex PTSD represented in the new diagnostic categories in DSM-5 and ICD-11 and emerging novel treatments [29].

### Generalizability of the results

In this study, we reported a first comprehensive dataset on clinicians’ treatment choices for PTSD among psychiatrists and psychiatry specialist trainees across Europe. This is particularly significant given that while the majority of participants correctly identified the diagnosis in the clinical case, there was a large variation in the preferred treatment options.

### Implications of the findings for future practice

The study has several important implications. First and foremost, it indicates a general convergence in the clinicians’ choices for pharmacotherapy (instead of psychotherapy) as treatment for PTSD across Europe, which is not anchored in current clinical guidelines, but is probably related to the availability of resources. The observed differences in specific pharmacological approaches (add-on medications) as well as psychotherapies between the four European regions call for more dialogue within the European national psychiatric associations and EPA community to promote standardization of best practice. Interestingly, we found that about half of the participants did not follow any specific guidelines when recommending treatment for PTSD. Therefore, the EPA should work to identify strategies to facilitate the implementation of guidelines to be endorsed by clinicians. The EPA will use these results to promote good clinical practice across Europe among mental health practitioners, mental health organizations, and European policy makers.

In line with this objective, the EPA should further promote and propose educational activities to support the implementation of best practice at different levels. This includes: 1) continuing and improving professional development for psychiatrists with a focus on clinical approaches to PTSD in different European regions; 2) harmonizing training programs and promoting standards of best practice by including them in the European Psychiatric Specialist Examination, which is currently under development; and 3) promoting real-world clinical trials and inclusion of the new findings in clinical guidelines.

## Supporting information

Rojnic Kuzman et al. supplementary material 1 Rojnic Kuzman et al. supplementary material

Rojnic Kuzman et al. supplementary material 2 Rojnic Kuzman et al. supplementary material

## Data Availability

The data that support the findings of this study are available as open source.

## Supplementary material

The supplementary material for this article can be found at http://doi.org/10.1192/j.eurpsy.2024.19.

## References

[1] Copeland WE, Shanahan L, Hinesley J, Chan RF, Aberg KA, Fairbank JA, et al. Association of childhood trauma exposure with adult psychiatric disorders and functional outcomes. JAMA Netw Open. 2018;1:e184493. doi:10.1001/jamanetworkopen.2018.4493. 30646356 PMC6324370

[2] Kubany ES, Leisen MB, Kaplan AS, Watson SB, Haynes SN, Owens JA, et al. Development and preliminary validation of a brief broad-spectrum measure of trauma exposure: the Traumatic Life Events Questionnaire. Psychol Assess. 2000;12:210–24. doi:10.1037/1040-3590.12.2.210. 10887767

[3] Morina N, Stam K, Pollet TV, Priebe S. Prevalence of depression and posttraumatic stress disorder in adult civilian survivors of war who stay in war-afflicted regions: a systematic review and meta-analysis of epidemiological studies. J Affect Disord. 2018;239:328–38. doi:10.1016/j.jad.2018.07.027 30031252

[4] Zeng N, Zhao Y-M, Yan W, Li C, Lu Q-D, Liu L, et al. A systematic review and meta-analysis of long term physical and mental sequelae of COVID-19 pandemic: call for research priority and action. Mol Psychiatry. 2023;28:423–33. doi:10.1038/s41380-022-01614-7 35668159 PMC9168643

[5] Chutiyami M, Cheong AMY, Salihu D, Bello UM, Ndwiga D, Maharaj R, et al. COVID-19 pandemic and overall mental health of healthcare professionals globally: a meta-review of systematic reviews. Front Psychiatry. 2022;12:804525. doi:10.3389/fpsyt.2021.804525 35111089 PMC8801501

[6] Hoell A, Kourmpeli E, Salize HJ, Heinz A, Padberg F, Habel U, et al. Prevalence of depressive symptoms and symptoms of post-traumatic stress disorder among newly arrived refugees and asylum seekers in Germany: systematic review and meta-analysis. BJPsych Open. 2021;7:e93. doi:10.1192/bjo.2021.54 33938425 PMC8142547

[7] Schäfer I, Hopchet M, Vandamme N, Ajdukovic D, El-Hage W, Egreteau L, et al. Trauma and trauma care in Europe. Eur J Psychotraumatol. 2018;9:1556553. doi:10.1080/20008198.2018.1556553 30637092 PMC6319458

[8] Hogg B, Gardoki-Souto I, Valiente-Gómez A, Rosa AR, Fortea L, Radua J, et al. Psychological trauma as a transdiagnostic risk factor for mental disorder: an umbrella meta-analysis. Eur Arch Psychiatry Clin Neurosci. 2023;273:397–410. doi:10.1007/s00406-022-01495-5 36208317

[9] UNHCR/WHO. Mental health of refugees. Geneva: UNHCR/WHO; 1996.

[10] Gupta MA. Review of somatic symptoms in post-traumatic stress disorder. Int Rev Psychiatry. 2013;25:86–99. doi:10.3109/09540261.2012.736367 23383670

[11] O’Donnell CJ, Schwartz Longacre L, Cohen BE, Fayad ZA, Gillespie CF, Liberzon I, et al. Posttraumatic stress disorder and cardiovascular disease. JAMA Cardiol. 2021;6:1207. doi:10.1001/jamacardio.2021.2530 34259831

[12] Kuzman MR, Slade M, Puschner B, Scanferla E, Bajic Z, Courtet P, et al. Clinical decision-making style preferences of European psychiatrists: results from the ambassadors survey in 38 countries. Eur Psychiatry. 2022;65:1–33. doi:10.1192/j.eurpsy.2022.2330 PMC9706307 36266742

[13] Semrau M, Barley E, Law A, Thornicroft G. Lessons learned in developing community mental health care in Europe. World Psychiatry. 2011;10:217–25. doi:10.1002/j.2051-5545.2011.tb00060.x 21991282 PMC3188777

[14] Winkler P, Krupchanka D, Roberts T, Kondratova L, Machů V, Höschl C, et al. A blind spot on the global mental health map: a scoping review of 25 years’ development of mental health care for people with severe mental illnesses in central and eastern Europe. Lancet Psychiatry. 2017;4:634–42. doi:10.1016/S2215-0366(17)30135-9 28495549

[15] National Institute for Health and Care Excellence (NICE). Post-traumatic stress disorder. NICE guideline [NG116]; 2018. Available from: https://www.nice.org.uk/guidance/ng116 (accessed December 1, 2023). 31211536

[16] Forbes D, Bisson JI, Monson CM, Berliner L. Effective treatments for PTSD. 3rd ed. Chicago, IL: Guilford Press; 2020.

[17] Burback L, Brémault-Phillips S, Nijdam MJ, McFarlane A, Vermetten E. Treatment of posttraumatic stress disorder: a state-of-the-art review. Curr Neuropharmacol. 2024;22:557–635. doi:10.2174/1570159X21666230428091433 37132142 PMC10845104

[18] Rojnic Kuzman M, Vahip S, Fiorillo A, Beezhold J, Pinto da Costa M, Skugarevsky O, et al. Mental health services during the first wave of the COVID-19 pandemic in Europe: results from the EPA ambassadors survey and implications for clinical practice. Eur Psychiatry. 2021;64:e41. doi:10.1192/j.eurpsy.2021.2215 34103102 PMC8314055

[19] World Medical Association. World Medical Association declaration of Helsinki: ethical principles for medical research involving human subjects. JAMA. 2013;310:2191–4. doi:10.1001/jama.2013.281053 24141714

[20] Vandenbroucke JP, von Elm E, Altman DG, Gøtzsche PC, Mulrow CD, Pocock SJ, et al. Strengthening the reporting of observational studies in epidemiology (STROBE): explanation and elaboration. Int J Surg. 2014;12:1500–24. doi:10.1016/j.ijsu.2014.07.014 25046751

[21] Schmitz A. Benzodiazepines: the time for systematic change is now. Addiction. 2021;116:219–21. doi:10.1111/add.15095 32335948

[22] Guina J, Rossetter SR, De Rhodes BJ, Nahhas RW, Welton RS. Benzodiazepines for PTSD. J Psychiatr Pract. 2015;21:281–303. doi:10.1097/PRA.0000000000000091 26164054

[23] Bushnell GA, Stürmer T, Gaynes BN, Pate V, Miller M. Simultaneous antidepressant and benzodiazepine new use and subsequent long-term benzodiazepine use in adults with depression, United States, 2001–2014. JAMA Psychiatry. 2017;74:747. doi:10.1001/jamapsychiatry.2017.1273 28593281 PMC5710248

[24] Maric NP, Andric Petrovic S, Russo M, Jerotic S, Ristic I, Savić B, et al. Maintenance therapy of psychosis Spectrum disorders in a real-world setting: antipsychotics prescription patterns and long-term benzodiazepine use. Front Psych. 2022;13:796719. doi:10.3389/fpsyt.2022.796719 PMC9022963 35463504

[25] World Health Organization. European Health for All database (HFA-DB). Eur Heal Inf Gatew; 2021. Available from: https://gateway.euro.who.int/en/datasets/european-health-for-all-database/ (accessed June 22, 2022).

[26] Fiorillo A, Luciano M, Giacco D, Del Vecchio V, Baldass N, De Vriendt N, et al. Training and practice of psychotherapy in Europe: results of a survey. World Psychiatry. 2011;10:238. doi:10.1002/j.2051-5545.2011.tb00064.x 21991286 PMC3188779

[27] Shapiro F. EMDR, adaptive information processing, and case conceptualization. J EMDR Pract Res. 2007;1:68–87. doi:10.1891/1933-3196.1.2.68

[28] European Union. EuroVoc multilingual thesaurus of the European Union; 2017. Available from: http://publications.europa.eu/resource/cellar/7eecbd11-c00d-11e5-9e54-01aa75ed71a1.0002.01/DOC_1 (accessed May 10, 2017).

[29] Mitchell JM, Ot’alora G. M, van der Kolk B, Shannon S, Bogenschutz M, Gelfand Y, et al. MDMA-assisted therapy for moderate to severe PTSD: a randomized, placebo-controlled phase 3 trial. Nat Med. 2023;29:2473–80. doi:10.1038/s41591-023-02565-4 37709999 PMC10579091

