# Supplementary material for: Clinician treatment choices for post-traumatic stress disorder: ambassadors survey of psychiatrists in 39 European countries
Source: Eur Psychiatry. 2024 Mar 7;67(1):e24. doi: 10.1192/j.eurpsy.2024.19 (PMC10988156; doi:10.1192/j.eurpsy.2024.19)
Supplement: Rojnic Kuzman et al. supplementary material 1 — Rojnic Kuzman et al. supplementary material [file S0924933824000191sup001.pdf]

EPA Ambassadors 2022

Following the successful surveys launched in 2020 and 2021 with more than 900 responses collected from all over Europe, the EPA is glad to announce the third edition of its Ambassadors Programme. Started in 2020, this initiative aims at engaging psychiatrists and mental health professionals to shape better mental health policies and programmes in Europe.

This year's questionnaire will focus on trauma-related diagnosis and treatment.

EPA Ambassadors 2022

Before we start...

Read the short paragraph below and click "Next" to agree and continue. Please note that the survey will require around 10 minutes to complete and your data will be analysed anonymously.

I agree to participate in a European survey led by the EPA (Ambassador wave 3), which collects information of my own profile/activity as a clinician, quoting two vignettes of patients with trauma, in order to analyse what would be my favoured diagnoses and treatments.

EPA Ambassadors 2022

* 1. Country where you work

* 2. Gender

* 3. Age

* 4. Profession

- [ ] Adult Psychiatrist
- [ ] Child and adolescent psychiatrist
- [ ] Psychiatric trainee
- [ ] Psychologist
- [ ] Other (please specify)

* 5. Setting

- [ ] University department
- [ ] Public hospital
- [ ] Private hospital
- [ ] Outpatient service
- [ ] Private practice
- [ ] Child and adolescent unit
- [ ] Community mental health
- [ ] Other (please specify)

* 6. How long have you worked as a mental health professional? (In years)

* 7. Are you a certified psychotherapist?

- ( ) No
- ( ) Yes

* 8. If you are a certified psychotherapist please select the main area of your work. (If you are not a psychotherapist select the option "not applicable")

* 9. Is psychotraumatology your main field of work?

- ◯ Yes
- ◯ No

* 10. For how many years? (type "0" if your previous response is "no")

* 11. How many patients with PTSD do you see in your clinical practice within a working month? (If you are unsure, please make an estimation)

* 12. What percentage of your working time you work in outpatient, inpatient or other settings? (Please note that the sum of the below responses should be "100").

Outpatients

Inpatients

Other settings

* 13. In your place of work, what trauma-focused therapy are you able to propose? (MULTIPLE ANSWERS ALLOWED)

- ☐ Trauma-focused CBT
- ☐ Prolonged exposure
- ☐ EMDR
- ☐ Psychodynamic oriented psychotherapy
- ☐ Narrative exposure Therapy
- ☐ Cognitive Processing Therapy
- ☐ Other trauma-focused psychotherapy
- ☐ None

* 14. In case you offer trauma-focused therapy, do you follow one or more of the below guidelines? (MULTIPLE ANSWERS ALLOWED)

- [ ] NICE
- [ ] APA
- [ ] WHO
- [ ] Veterans Affairs and US Department of Defense
- [ ] ISTSS
- [ ] National guidelines
- [ ] No specific guidelines are implemented

Cases

Please read the two typical cases of reactions to trauma and respond to the questions related to each case based on your experience from the clinical practice at the PLACE OF YOUR FORMAL WORK.

CASE

A 35-year old shop manager from Kyiv has arrived at your place of work after she had developed a depressed mood, insomnia and “flashbacks” related to her experiences of wartime. She left her country together with her two young children 3 months ago and started to have symptoms 4 weeks after arriving in the present country. She became particularly concerned with watching TV news, and tried to avoid talking to other refugees and leaving the flat. She also felt eventually guilty for surviving. She became tense, felt readily emotionally overwhelmed, and more and more distant from her children.

* 15. What would most likely be the main diagnosis for a patient with the above symptoms?

- ◯ Adjustment disorder
- ◯ Acute stress reaction
- ◯ PTSD
- ◯ Anxiety disorder
- ◯ Depression
- ◯ Other
- ◯ I don’t know

* 16. How confident are you about your own diagnosis in the above mentioned patient? (Where 0% is "not confident at all" and 100% represents "absolutely confident")

[ ]

* 17. Would you apply validated self-report and interview measures? If yes, which ones of the following? (MULTIPLE ANSWERS ALLOWED)

- ☐ Global Assessment of Posttraumatic Stress Scale 5 (EGEP-5)
- ☐ Clinical-Administered PTSD Scale (CAPS)
- ☐ The Complex Trauma Questionnaire (CTQ)
- ☐ The International Trauma Questionnaire (ITQ)
- ☐ PTSD Symptom Scale Interview (PSS-I)
- ☐ Structured Clinical Interview; PTSD Module (SCID PTSD Module)
- ☐ Hamilton Depression Rating Scale (HDRS)
- ☐ Montgomery-Asberg Depression Rating Scale (MADRS)
- ☐ Hamilton Anxiety Rating Scale (HAM-A)
- ☐ None of these, but I would use some
- ☐ I would not apply any scale

* 18. Would you consider checking for somatic disorders or referring the described patient? (MULTIPLE ANSWERS ALLOWED)

- [ ] No
- [ ] Yes, I will take anamnestic data on the existence of somatic disorders
- [ ] Yes, I would refer the patient to consultations from internal medicine specialists
- [ ] Yes, ask for laboratory measurements for metabolic syndrome
- [ ] Yes, ask for specific screening for cardiovascular disorders for example ECG, blood pressure
- [ ] Yes, general laboratory measures (for example blood count)

* 19. How would you treat this patient? (MULTIPLE ANSWERS ALLOWED)

- [ ] Venlafaxine
- [ ] Sertraline
- [ ] Other SSRI
- [ ] Trazodone
- [ ] Mirtazapine
- [ ] Tricyclic antidepressant
- [ ] Benzodiazepine
- [ ] Anticonvulsants
- [ ] Antipsychotics
- [ ] Cognitive Behavioural Therapy
- [ ] Psychodynamic Psychotherapy
- [ ] Systemic Therapy
- [ ] Trauma-focused CBT
- [ ] Prolonged exposure
- [ ] EMDR
- [ ] Narrative exposure Therapy
- [ ] Cognitive Processing Therapy
- [ ] Other trauma-focused psychotherapy
- [ ] Psychoeducation about trauma
- [ ] Other (please specify)

* 20. For this case, would you suggest group treatment? Or individual treatment?

- ○ Group treatment
- ○ Individual treatment
- ○ Both

* 21. What treatment setting would you recommend in these cases?

- ◯ Hospital treatment
- ◯ Outpatient treatment
- ◯ Daily hospital treatment
- ◯ Community mental health teams

* 22. Who would most likely provide the treatment in such a case? (Please mark the closest response to your opinion)

- ◯ I would treat this patient myself
- ◯ I would involve colleagues specialized for trauma in my workplace
- ◯ I would refer them to a specialized centre for trauma
- ◯ I would refer them to a specialized centre for specific type of trauma or group of traumatized persons (i.e. working with women only)
- ◯ I would refer them to the GP for follow up after the initial diagnosis

* 23. What is the expected duration of treatment in these cases (in months)?

* 24. Would you involve other persons/sectors in the treatment? (MULTIPLE ANSWERS ALLOWED)

- ☐ No
- ☐ Peer groups
- ☐ Family members
- ☐ Social workers
- ☐ Occupational therapist
- ☐ Employment service
- ☐ Professional rehabilitation service
- ☐ Professional translator
- ☐ Non-professional translator
- ☐ Other (please specify)

* 25. Do you have access to a translator, if needed?

- ◯ Yes
- ◯ No
- ◯ I do not know

* 26. If you involve a translator, is she/he reimbursed?

- Yes
- No
- I do not know

* 27. How many migrants from Ukraine have you worked with in your role as mental health professional in the last working month? (If you are unsure, please estimate)

CASE

A 33 -year-old bank manager from the main city of your country has arrived at your place of work after she had developed depressed mood, insomnia and "flashbacks" related to a robbery where her colleague was killed at her work place 3 months ago. The symptoms have started within the first 4 weeks after this event. Soon she became particularly concerned with going to work, but also watching news on TV, where she had recently seen a documentary on this event. She became anxious, felt guilty, emotionally overwhelmed and more and more distant from her two young children.

* 28. What would most likely be the main diagnosis for a patient with the above symptoms?

- ◯ Adjustment disorder
- ◯ Acute stress reaction
- ◯ PTSD
- ◯ Anxiety disorder
- ◯ Depression
- ◯ Other
- ◯ I don't know

* 29. How confident are you about your own diagnosis in the above mentioned patient? (Where 0% is "not confident at all" and 100% represents "absolutely confident")

[ ]

* 30. Would you apply validated self-report and interview measures? If yes, which ones of the following? (MULTIPLE ANSWERS ALLOWED)

- ☐ Global Assessment of Posttraumatic Stress Scale 5 (EGEP-5)
- ☐ Clinical-Administered PTSD Scale (CAPS)
- ☐ The Complex Trauma Questionnaire (CTQ)
- ☐ The International Trauma Questionnaire (ITQ)
- ☐ PTSD Symptom Scale Interview (PSS-I)
- ☐ Structured Clinical Interview; PTSD Module (SCID PTSD Module)
- ☐ Hamilton Depression Rating Scale (HDRS)
- ☐ Montgomery-Asberg Depression Rating Scale (MADRS)
- ☐ Hamilton Anxiety Rating Scale (HAM-A)
- ☐ None of these but I would use some
- ☐ I would not apply any scale

* 31. Would you consider checking for somatic disorders or referring the described patient? (MULTIPLE ANSWERS ALLOWED)

- [ ] No
- [ ] Yes, I will take anamnestic data on the existence of somatic disorders
- [ ] Yes, I would refer the patient to consultations from internal medicine specialists
- [ ] Yes, ask for laboratory measurements for metabolic syndrome
- [ ] Yes, ask for specific screening for cardiovascular disorders for example ECG, blood pressure
- [ ] Yes, general laboratory measures (for example blood count)

* 32. How would you treat this patient? (MULTIPLE ANSWERS ALLOWED)

- [ ] Venlafaxine
- [ ] Sertraline
- [ ] Other SSRI
- [ ] Trazodone
- [ ] Mirtazapine
- [ ] Tricyclic antidepressant
- [ ] Benzodiazepine
- [ ] Anticonvulsants
- [ ] Antipsychotics
- [ ] Cognitive Behavioural Therapy
- [ ] Psychodynamic Psychotherapy
- [ ] Systemic Therapy
- [ ] Trauma-focused CBT
- [ ] Prolonged exposure
- [ ] EMDR
- [ ] Narrative exposure Therapy
- [ ] Cognitive Processing Therapy
- [ ] Other trauma-focused psychotherapy
- [ ] Psychoeducation about trauma
- [ ] Other (please specify)

* 33. For this case, would you suggest group treatment? Or individual treatment?

- ○ Group treatment
- ○ Individual treatment
- ○ Both

* 34. What treatment setting would you recommend in these cases?

- ○ Hospital treatment
- ○ Outpatient treatment
- ○ Daily hospital treatment
- ○ Community mental health teams

* 35. Who would most likely provide the treatment in such a case? (Please mark the closest response to your opinion)

- ○ I would treat this patient myself
- ○ I would involve colleagues specialized for trauma in my workplace
- ○ I would refer them to a specialized centre for trauma
- ○ I would refer them to a specialized centre for specific type of trauma or group of traumatized persons (i.e. working with women only)
- ○ I would refer them to the GP for follow up after the initial diagnosis

* 36. What is the expected duration of treatment in these cases (in months)?

* 37. Would you involve other persons/sectors in the treatment? (MULTIPLE ANSWERS ALLOWED)

- ☐ No
- ☐ Peer groups
- ☐ Family members
- ☐ Social workers
- ☐ Occupational therapist
- ☐ Employment service
- ☐ Professional rehabilitation service
- ☐ Professional translator
- ☐ Non-professional translator
- ☐ Other (please specify)

* 38. Do you have access to a translator, if needed?

- ○ Yes
- ○ No
- ○ I do not know

* 39. If you involve a translator, is she/he reimbursed?

- Yes
- No
- I do not know

EPA Ambassadors 2022

* 40. Do you agree to participate in future Ambassadors activities?

- Yes
- No

41. If yes, please provide your email address:
