# Supplementary material for: Clinician treatment choices for post-traumatic stress disorder: ambassadors survey of psychiatrists in 39 European countries
Source: Eur Psychiatry. 2024 Mar 7;67(1):e24. doi: 10.1192/j.eurpsy.2024.19 (PMC10988156; doi:10.1192/j.eurpsy.2024.19)
Supplement: Rojnic Kuzman et al. supplementary material 2 — Rojnic Kuzman et al. supplementary material [file S0924933824000191sup002.docx]

**SUPPLEMENTARY MATERIALS**

**Supplementary Table 1.** Country by region; only European psychiatrists and psychiatry residents; raw, unweighted data; total sample (n = 948)

|  | CE Europe  (n = 435) | Southern  (n = 136) | Northern  (n = 143) | Western  (n = 234) | Total  (n = 948) |
| --- | --- | --- | --- | --- | --- |
| Croatia | 43 (9.9) |  |  |  | 43 (4.5) |
| Russia | 39 (9.0) |  |  |  | 39 (4.1) |
| Serbia | 34 (7.8) |  |  |  | 34 (3.6) |
| Poland | 29 (6.7) |  |  |  | 29 (3.1) |
| Georgia | 28 (6.4) |  |  |  | 28 (3.0) |
| Belarus | 24 (5.5) |  |  |  | 24 (2.5) |
| North Macedonia | 24 (5.5) |  |  |  | 24 (2.5) |
| Azerbaijan | 23 (5.3) |  |  |  | 23 (2.4) |
| Slovakia | 22 (5.1) |  |  |  | 22 (2.3) |
| Bosnia and Herzegovina | 21 (4.8) |  |  |  | 21 (2.2) |
| Czech Republic | 21 (4.8) |  |  |  | 21 (2.2) |
| Ukraine | 20 (4.6) |  |  |  | 20 (2.1) |
| Hungary | 19 (4.4) |  |  |  | 19 (2.0) |
| Slovenia | 19 (4.4) |  |  |  | 19 (2.0) |
| Bulgaria | 17 (3.9) |  |  |  | 17 (1.8) |
| Moldova | 17 (3.9) |  |  |  | 17 (1.8) |
| Romania | 16 (3.7) |  |  |  | 16 (1.7) |
| Montenegro | 14 (3.2) |  |  |  | 14 (1.5) |
| Armenia | 5 (1.1) |  |  |  | 5 (0.5) |
| Spain |  | 43 (31.6) |  |  | 43 (4.5) |
| Turkey |  | 39 (28.7) |  |  | 39 (4.1) |
| Portugal |  | 30 (22.1) |  |  | 30 (3.2) |
| Italy |  | 22 (16.2) |  |  | 22 (2.3) |
| Greece |  | 2 (1.5) |  |  | 2 (0.2) |
| Finland |  |  | 82 (57.3) |  | 82 (8.6) |
| Latvia |  |  | 19 (13.3) |  | 19 (2.0) |
| Lithuania |  |  | 19 (13.3) |  | 19 (2.0) |
| Estonia |  |  | 13 (9.1) |  | 13 (1.4) |
| Sweden |  |  | 5 (3.5) |  | 5 (0.5) |
| Norway |  |  | 3 (2.1) |  | 3 (0.3) |
| Denmark |  |  | 2 (1.4) |  | 2 (0.2) |
| United Kingdom |  |  |  | 78 (33.3) | 78 (8.2) |
| Ireland |  |  |  | 38 (16.2) | 38 (4.0) |
| Belgium |  |  |  | 32 (13.7) | 32 (3.4) |
| Switzerland |  |  |  | 32 (13.7) | 32 (3.4) |
| Germany |  |  |  | 25 (10.7) | 25 (2.6) |
| France |  |  |  | 24 (10.3) | 24 (2.5) |
| Netherlands |  |  |  | 3 (1.3) | 3 (0.3) |
| Austria |  |  |  | 2 (0.9) | 2 (0.2) |

European regions were defined according to the EU Vocabularies [30]**.** Data are presented as numbers (percentages) of participants if not stated otherwise.

**Supplementary Table 2.** Country by region; only European psychiatrists and psychiatry residents who answered case 2 and identified PTSD as the most likely diagnosis in the case 2; raw, unweighted data (n = 611)

|  | CE Europe  (n = 279) | Southern  (n = 92) | Northern  (n = 92) | Western  (n = 148) | Total  (n = 611) |
| --- | --- | --- | --- | --- | --- |
| Serbia | 25 (9.0) |  |  |  | 25 (4.1) |
| Croatia | 24 (8.6) |  |  |  | 24 (3.9) |
| Russia | 21 (7.5) |  |  |  | 21 (3.4) |
| North Macedonia | 19 (6.8) |  |  |  | 19 (3.1) |
| Azerbaijan | 17 (6.1) |  |  |  | 17 (2.8) |
| Georgia | 17 (6.1) |  |  |  | 17 (2.8) |
| Slovakia | 17 (6.1) |  |  |  | 17 (2.8) |
| Bulgaria | 16 (5.7) |  |  |  | 16 (2.6) |
| Ukraine | 16 (5.7) |  |  |  | 16 (2.6) |
| Czech Republic | 15 (5.4) |  |  |  | 15 (2.5) |
| Poland | 15 (5.4) |  |  |  | 15 (2.5) |
| Belarus | 13 (4.7) |  |  |  | 13 (2.1) |
| Hungary | 12 (4.3) |  |  |  | 12 (2.0) |
| Montenegro | 12 (4.3) |  |  |  | 12 (2.0) |
| Bosnia and Herzegovina | 11 (3.9) |  |  |  | 11 (1.8) |
| Romania | 10 (3.6) |  |  |  | 10 (1.6) |
| Slovenia | 10 (3.6) |  |  |  | 10 (1.6) |
| Moldova | 8 (2.9) |  |  |  | 8 (1.3) |
| Armenia | 1 (0.4) |  |  |  | 1 (0.2) |
| Spain |  | 29 (31.5) |  |  | 29 (4.7) |
| Turkey |  | 27 (29.3) |  |  | 27 (4.4) |
| Portugal |  | 20 (21.7) |  |  | 20 (3.3) |
| Italy |  | 14 (15.2) |  |  | 14 (2.3) |
| Greece |  | 2 (2.2) |  |  | 2 (0.3) |
| Finland |  |  | 58 (63.0) |  | 58 (9.5) |
| Latvia |  |  | 10 (10.9) |  | 10 (1.6) |
| Lithuania |  |  | 9 (9.8) |  | 9 (1.5) |
| Estonia |  |  | 8 (8.7) |  | 8 (1.3) |
| Sweden |  |  | 4 (4.3) |  | 4 (0.7) |
| Denmark |  |  | 2 (2.2) |  | 2 (0.3) |
| Norway |  |  | 1 (1.1) |  | 1 (0.2) |
| United Kingdom | |  |  | 52 (35.1) | 52 (8.5) |
| Ireland |  |  |  | 19 (12.8) | 19 (3.1) |
| Germany |  |  |  | 16 (10.8) | 16 (2.6) |
| France |  |  |  | 17 (11.5) | 17 (2.8) |
| Belgium |  |  |  | 21 (14.2) | 21 (3.4) |
| Switzerland |  |  |  | 21 (14.2) | 21 (3.4) |
| Austria |  |  |  | 1 (0.7) | 1 (0.2) |
| Netherlands |  |  |  | 1 (0.7) | 1 (0.2) |

European regions were defined according to the EU Vocabularies [30]**.** Data are presented as numbers (percentages) of participants if not stated otherwise.

**Supplementary Table 3.** Differences between respondents who did and did not identify PTSD as the most likely diagnosis in the vignette; only European psychiatrists and psychiatry residents; raw, unweighted data; total sample (n = 948)

|  | Identified PTSD | |  |  |
| --- | --- | --- | --- | --- |
|  | Yes  (n = 611) | No  (n = 198) | Missing data  (n = 139) | p |
| Region |  |  |  |  |
| CE | 279 (45.7) | 103 52.0) | 53 (38.1) | 0.197 |
| Southern | 92 (15.1) | 26 (13.1) | 18 (12.0) |  |
| Northern | 92 (15.1) | 24 (12.1) | 27 (19.4) |  |
| Western | 148 (24.2) | 45 (22.7) | 41 (29.5) |  |
| Age (years), mean (SD) | 46 (12) | 47 (12) | 47 (14) | 0.943 |
| Gender |  |  |  |  |
| men | 269 (44.0) | 95 (48.0) | 47 (33.8) | 0.030 |
| women | 342 (56.0) | 103 (52.0) | 92 (66.2) |  |
| Professional status |  |  |  |  |
| residents | 72 (11.8) | 22 (11.1) | 19 (13.7) | 0.764 |
| psychiatrists | 539 (88.2) | 176 (88.9) | 120 (86.3) |  |
| Workplace |  |  |  |  |
| University department | 236 (38.6) | 70 (35.4) | 43 (30.9) | 0.211 |
| Public hospital | 245 (40.1) | 90 (45.5) | 59 (42.4) | 0.403 |
| Private hospital or private practice | 153 (25.0) | 45 (22.7) | 40 (28.8) | 0.451 |
| Community mental health | 63 (10.3) | 14 (7.1) | 14 (10.1) | 0.396 |
| Work experience (years), mean (SD) | 18 (11) | 18 (12) | 18 (13) | 0.918 |
| Certified psychotherapists | 258 (42.2) | 71 (35.9) | 65 (46.0) | 0.142 |
| Certified psychotherapists main area |  |  |  |  |
| Cognitive behavioural therapy | 98 (38.7) | 28 (38.4) | 24 (41.4) | 0.569 |
| Psychodynamic oriented | 25 (9.9) | 4 (5.5) | 8 (13.8) |  |
| Integrative psychotherapy | 16 (6.3) | 11 (15.1) | 3 (5.2) |  |
| Systemic family therapy | 14 (5.5) | 4 (5.5) | 6 (10.3) |  |
| Psychoanalytic psychotherapy | 35 (13.8) | 8 (11.0) | 6 (10.3) |  |
| EMDR | 34 (13.4) | 7 (9.6) | 3 (5.2) |  |
| Other | 31 (12.3) | 11 (15.1) | 8 (13.8) |  |
| Posttraumatologists | 107 (17.5) | 28 (14.1) | 18 (12.9) | 0.289 |
| PTSD patients monthly, mean (SD) | 7 (11) | 7 (19) | 8 (15) | 0.918 |
| Availability of trauma-focused therapies |  |  |  |  |
| Trauma-focused CBT | 282 (46.2) | 79 (39.9) | 52 (37.4) | 0.087 |
| EMDR | 52 (8.5) | 22 (11.1) | 31 (22.3) | 0.004 |
| Psychodynamic oriented | 190 (31.1) | 40 (20.2) | 33 (23.7) | 0.389 |
| Cognitive processing therapy | 176 (28.8) | 60 (30.3) | 17 (12.2) | 0.024 |
| Narrative exposure therapy | 61 (10.0) | 17 (8.6) | 11 (7.9) | 0.684 |
| Prolonged exposure | 132 (21.6) | 47 (23.7) | 6 (4.3) | 0.087 |
| Other trauma-focused psychotherapy | 125 (20.5) | 33 (16.7) | 21 (15.1) | 0.232 |
| No trauma-focused therapy | 142 (23.2) | 53 (26.8) | 45 (32.4) | 0.072 |
| Guidelines followed |  |  |  |  |
| National guidelines | 166 (27.2) | 53 (26.8) | 36 (25.9) | 0.954 |
| NICE | 161 (26.4) | 49 (24.7) | 23 (16.5) | 0.053 |
| WHO | 103 (16.9) | 36 (18.2) | 19 (13.7) | 0.537 |
| APA | 74 (12.1) | 25 (12.6) | 17 (12.2) | 0.982 |
| ISTSS | 28 (4.6) | 5 (2.5) | 3 (2.2) | 0.231 |
| U.S. Department of Defence | 13 (2.1) | 5 (2.5) | 3 (2.2) | 0.946 |
| No specific guidelines | 306 (50.1) | 101 (51.0) | 85 (61.2) | 0.060 |

Data are presented as numbers (percentages) of participants if not stated otherwise.

**Supplementary Figure 1.** Share in population and sample; only European psychiatrists and psychiatry residents, total sample (n = 948); European regions were defined according to the EU Vocabularies [30]

**Supplementary Figure 2**. Participants flow chart

**Supplementary Table 4.** Fit indices for six models with different number of latent classes

| Number of  latent classes | LL | Np | CAIC | BIC | SABIC | BF |
| --- | --- | --- | --- | --- | --- | --- |
| 1 | -4440 | 15 | 8991 | 8976 | 8968 | - |
| 2 | -4141 | 29 | 8498 | 8469 | 8453 | 0.00 |
| 3 | -3929 | 43 | 8177 | 8134 | 8108 | 0.00 |
| 4 | -3738 | 53 | 7870* | 7817* | 7784* | 0.00* |
| 5 | -3738 | 64 | 7952 | 7888 | 7848 | 34.09 |
| 6 | -3671 | 87 | 7986 | 7899 | 7845 | 1.79 |

Abbreviations: LL, log-likelihood; np, number of estimated parameters; CAIC, Consistent Akaike information criterion; BIC, Bayesian information criterion; SABIC, Sample size adjusted BIC; BF, Bayes factor

* The model with the best fit to empirical data
